# Incomplete Deletion of IL-4Rα by LysM^Cre^ Reveals Distinct Subsets of M2 Macrophages Controlling Inflammation and Fibrosis in Chronic Schistosomiasis

**DOI:** 10.1371/journal.ppat.1004372

**Published:** 2014-09-11

**Authors:** Kevin M. Vannella, Luke Barron, Lee A. Borthwick, Kristen N. Kindrachuk, Prakash Babu Narasimhan, Kevin M. Hart, Robert W. Thompson, Sandra White, Allen W. Cheever, Thirumalai R. Ramalingam, Thomas A. Wynn

**Affiliations:** 1 Program in Tissue Immunity and Repair, Laboratory of Parasitic Diseases, National Institute of Allergy and Infectious Diseases, National Institutes of Health, Bethesda, Maryland, United States of America; 2 Tissue Fibrosis and Repair Group, Institute of Cellular Medicine, Newcastle University, Newcastle upon Tyne, United Kingdom; 3 Biomedical Research Institute, Rockville, Maryland, United States of America; Case Western Reserve University, United States of America

## Abstract

Mice expressing a Cre recombinase from the lysozyme M-encoding locus (*Lyz2*) have been widely used to dissect gene function in macrophages and neutrophils. Here, we show that while naïve resident tissue macrophages from IL-4Rα^flox/delta^LysM^Cre^ mice almost completely lose IL-4Rα function, a large fraction of macrophages elicited by sterile inflammatory stimuli, *Schistosoma mansoni* eggs, or *S. mansoni* infection, fail to excise *Il4rα*. These F4/80^hi^CD11b^hi^ macrophages, in contrast to resident tissue macrophages, express lower levels of *Lyz2* explaining why this population resists LysM^Cre^-mediated deletion. We show that in response to IL-4 and IL-13, Lyz2^lo^IL-4Rα^+^ macrophages differentiate into an arginase 1-expressing alternatively-activated macrophage (AAM) population, which slows the development of lethal fibrosis in schistosomiasis. In contrast, we identified Lyz2^hi^IL-4Rα^+^ macrophages as the key subset of AAMs mediating the downmodulation of granulomatous inflammation in chronic schistosomiasis. Our observations reveal a limitation on using a LysM^Cre^ mouse model to study gene function in inflammatory settings, but we utilize this limitation as a means to demonstrate that distinct populations of alternatively activated macrophages control inflammation and fibrosis in chronic schistosomiasis.

## Author Summary

Chronic injury and inflammation lead to irreversible fibrosis in a range of diseases and infections. Macrophages alternatively activated by the immune system are capable of regulating inflammation and fibrosis, but our understanding of the source and function of these cells is incomplete. Mice genetically engineered to specifically prevent macrophages from becoming alternatively activated have been used to study the cells' role following infection with the parasite, *Schistosoma mansoni*. To our surprise, we found these mice prevent alternative activation only in macrophages that have had time to mature and some, perhaps more nascent, macrophages can become alternatively activated following exposure to *S. mansoni* eggs. We detected lower expression of *Lyz2* gene in these cells, leading to less expression of the enzyme excising the receptor gene necessary for alternative activation. Following *S. mansoni* infection, the livers of these mice have similar levels of fibrosis but significantly more inflammation compared to controls. We conclude that during schistosomiasis, distinct populations of alternatively activated macrophages control inflammation and fibrosis: macrophages expressing low levels of *Lyz2* express Arg1 and thus are sufficient to control fibrosis, while more mature *Lyz2*-expressing macrophages are required for downmodulation of egg-induced inflammation in chronic schistosomiasis.

## Introduction

Tissue macrophages exhibit substantial plasticity and can quickly change their function in response to different stimuli found in the local milieu [1], and distinct subsets with characteristic functional activities have been described. Alternatively activated macrophages (AAMs), also called M2 or M(IL-4) [2], are induced in response to the type-2 cytokines IL-4 and IL-13 [3], exhibit potent immunoregulatory activity, and have been linked with mechanisms controlling wound healing and fibrosis [4]. In addition to expressing mediators that directly regulate wound repair pathways such as arginase 1 (Arg1), resistin-like molecule alpha (Relm-α), transforming growth factor beta-1 (TGF-β1), vascular endothelial growth factor (VEGF), and insulin-like growth factor-1 (IGF-1) [5], AAMs also suppress pro-inflammatory Th1, Th17, and classically activated macrophage (CAMs) responses that contribute to tissue injury [6].

To prevent alternative activation, Herbert and colleagues generated macrophage/neutrophil-specific IL-4Rα-deficient mice (IL-4Rα^flox/Δ^LysM^Cre^) by expressing Cre recombinase in the regulatory region of the lysozyme M gene expressed in macrophages and neutrophils. They showed AAMs are required to suppress pathogenic Th1/CAM responses during infection with the helminth parasite *Schistosoma mansoni* [7]. However, AAMs had no significant impact on the development of the Th2 response or fibrosis.

In contrast to IL-4Rα^flox/Δ^LysM^Cre^ mice, mice with a macrophage/neutrophil-specific deletion of Arg1 (Arg1^flox/Δ^LysM^Cre^), an enzyme involved in the conversion of L-arginine into L-ornithine and urea, developed enhanced type-2 effector responses following *S. mansoni* infection without acute type-1 cytokine-driven hepatotoxicity or endotoxemia [8]. Pesce *et al*. observed Arg1^flox/Δ^LysM^Cre^ mice developed stronger CD4^+^ Th2 cell responses, larger eosinophil-rich granulomas, more severe liver fibrosis, and failed to down-regulate the type-2 inflammatory response when chronically infected, suggesting that Arg1^+^ macrophages critically suppress granulomatous inflammation, fibrosis, and mortality [9]. Similar but even more dramatic findings were observed with Arg1^flox/flox^Tie2^Cre^ mice, which delete Arg1 in all macrophage populations [8].

The ability of IL-4Rα^flox/Δ^LysM^Cre^ mice to control fibrosis during *S. mansoni* infection was completely unexpected, since Arg1 expression in macrophages was thought to be highly dependent on IL-4Rα signaling [7]. Because it was concluded that IL-4Rα-expressing AAMs suppress lethal type-1-associated inflammation during acute schistosomiasis, while arginase 1-expressing AAMs are dispensable during the acute stage, we theorized that an IL-4Rα-dependent but arginase 1-independent mechanism was responsible for the early protective activity exhibited by AAMs. To identify this mechanism, we set out to compare pathology, fibrosis, and the macrophage phenotype of IL-4Rα^flox/Δ^LysM^Cre^ mice and Arg1^flox/Δ^LysM^Cre^ mice following *S. mansoni* infection. We began by systematically studying IL-4Rα^flox/Δ^LysM^Cre^ mice during acute and chronic infection, and unexpectedly, we identified a subset of IL-4Rα-expressing macrophages that are resistant to LysM^Cre^-mediated gene deletion, exhibited an Arg1^+^ AAM phenotype, and regulated type-2 cytokine-dependent fibrosis. In contrast, we identified mature Lyz2^hi^ tissue macrophages that are susceptible to LysM^Cre^-mediated gene deletion as the critical population of AAMs mediating the downmodulation of granuloma formation in chronic schistosomiasis. So while these data suggest that the LysM^Cre^ deleter mouse is only useful for studying gene function in mature tissue macrophages, we were able to demonstrate that distinct populations of Lyz2^hi^ and Lyz2^lo^ AAMs collaborate to control inflammation and fibrosis in schistosomiasis, respectively. In chronic inflammatory diseases where the recruitment of immature monocytes/macrophages, differentiation to mature tissue macrophages, and acquisition of the alternatively activated phenotype is a dynamic and ongoing process, LysM^Cre^ can be used to distinguish the contribution of genes expressed in Lyz2^hi^ and Lyz2^lo^ macrophages.

## Results

### Surviving chronic *S. mansoni* infection depends on *Il4rα* allele, not LysM^Cre^ expression

**Figure 1:**
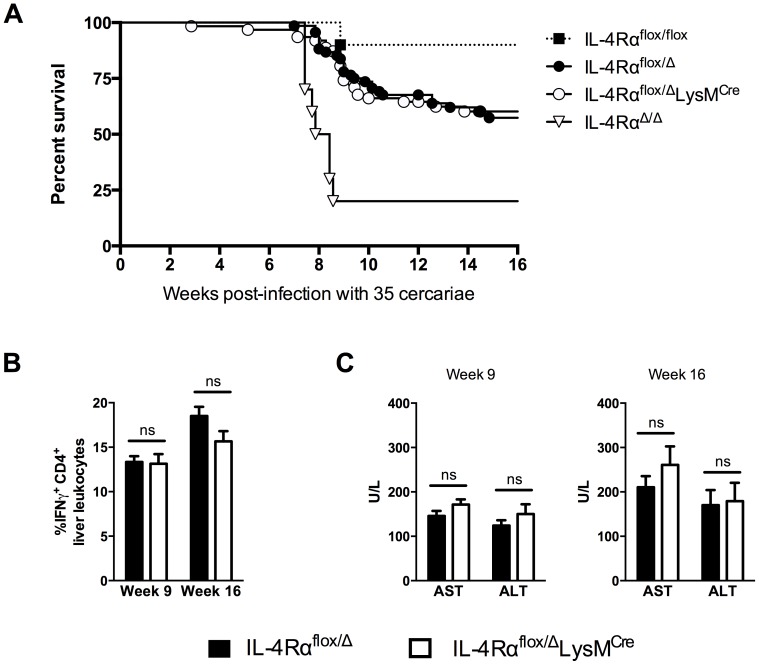
Surviving chronic *S. mansoni* infection depends on *Il4rα* allele, not LysM^Cre^ expression. IL-4Rα^flox/Δ^LysM^Cre^ mice (open circles and bars), IL-4Rα^flox/Δ^ littermate controls (solid circles and bars), IL-4Rα^flox/flox^ mice (solid squares), and IL-4Rα^Δ/Δ^ (open triangles) were infected percutaneously with 35 *Schistosoma mansoni* cercariae. A. Survival kinetics through 16 weeks (n = 10–20 per group). B. Th1 response. 9 or 16 weeks post-infection, liver leukocytes were isolated, restimulated with phorbol myristate acetate/ionomycin, stained for IFN-γ, and analyzed by flow cytometry (n = 7–15, ns = not significant). C. Hepatotoxicity. Aspartate aminotransferase (AST) and alanine aminotransferase (ALT) were assayed in serum 9 or 16 weeks after infection (n = 7–8 mice, ns = not significant). Data shown are mean ±SEM and represent at least two independent experiments.

The previous study by Herbert *et al*. found nearly 100% of IL-4Rα^flox/Δ^LysM^Cre^ mice succumbed to infection by 8 weeks post-infection [7]. Those experiments were conducted with 75–100 *S. mansoni* cercariae, a relatively high dose of parasites. To further elucidate the role of AAMs during a chronic *S. mansoni* infection and to explore the role of IL-4Rα-expressing macrophages in the initiation and regulation of fibrosis, we infected IL-4Rα^flox/Δ^LysM^Cre^ mice and IL-4Rα^flox/Δ^ littermate control mice with 35 cercariae, a dose that in wild-type mice leads to substantial disease and liver fibrosis but low mortality through the chronic phase of infection [10]. We hypothesized that lighter infections would enable us to quantify fibrosis, characterize the immune response, and phenotype the macrophage response in the granulomatous liver at both acute (9 weeks post-infection) and chronic (16 weeks post-infection) time-points. We observed 30–40% of the infected littermate control group (IL-4Rα^flox/Δ^) died through week 16 of infection (**Fig. 1A**). Surprisingly, we observed equal mortality in the IL-4Rα^flox/Δ^LysM^Cre^ group, suggesting that AAMs might be less important to survival in schistosomiasis than previously thought. The majority of the deaths occurred during the acute phase of the infection when the host immune response peaks and before additional protective mechanisms like IL-10 or the IL-13 decoy receptor (IL-13Rα2) are fully activated [11]. After week 10 of infection, few deaths were observed in either group. In the prior study, IL-4Rα^flox/Δ^LysM^Cre^ mice infected with ≥75 cercariae developed hepatotoxicity and gut pathology leading to endotoxemia and death. The authors also observed a stronger type-1 immune response in the IL-4Rα^flox/Δ^LysM^Cre^ mice, defined by increased IFN-γ production, which they hypothesized was contributing to the rapid death of the mice. Intriguingly, in our studies with IL-4Rα^flox/Δ^LysM^Cre^ mice infected with fewer cercariae, we observed no increase in IFN-γ (**Fig. 1B**) or hepatotoxicity at either acute or chronic time points (**Fig. 1C**). Importantly, we included wild-type IL-4Rα^flox/flox^ mice and IL-4Rα^Δ/Δ^ mice in the survival study to demonstrate universal deletion of IL-4Rα on one chromosome is the explanation for the enhanced mortality of both flox/Δ cohorts (**Fig. 1A**). As expected, mice with two copies deleted (Δ/Δ), showed the most susceptibility. We confirmed the infectious burdens were not different between the groups (**Fig. S1**). Because this finding was unexpected, we also tested a higher infectious dose. While an increase from 35 to 100 cercariae accelerated and significantly increased mortality in both groups, there was still no significant difference in mortality between the IL-4Rα^flox/Δ^LysM^Cre^ mice and IL-4Rα^flox/Δ^ littermate controls (**Fig. S2**).

### Inflammation but not fibrosis was exacerbated in chronically infected IL-4Rα^flox/Δ^LysM^Cre^ mice

**Figure 2:**
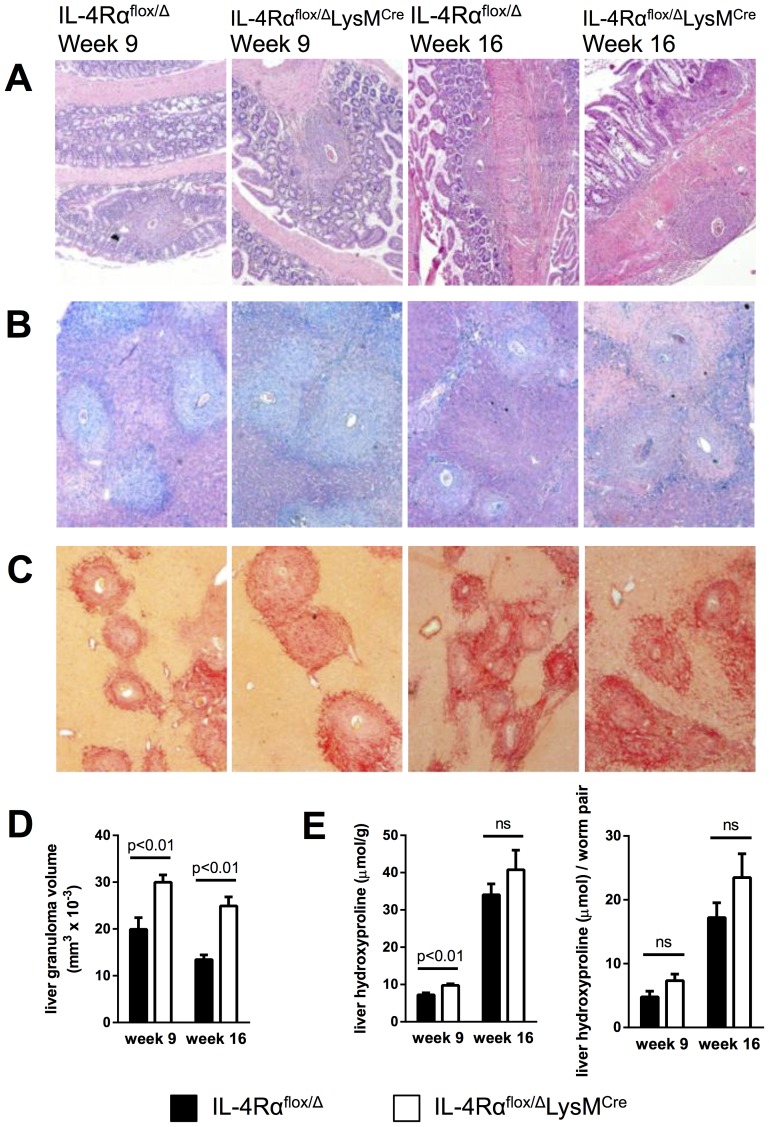
Inflammation but not fibrosis is exacerbated in chronically infected IL-4Rα^flox/Δ^LysM^Cre^ mice. IL-4Rα^flox/Δ^LysM^Cre^ mice and IL-4Rα^flox/Δ^ littermate controls were infected percutaneously with 35 *S. mansoni* cercariae. A–C. Representative 10× images of granuloma formation 9 weeks and 16 weeks post-infection from (A) hematoxylin and eosin-stained sections of intestinal tissue, (B) Giemsa-stained sections of liver tissue, or (C) picrosirius red-stained sections of liver tissue. D. Liver granuloma size in IL-4Rα^flox/Δ^LysM^Cre^ (open bars) and IL-4Rα^flox/Δ^ littermate control (solid bars) mice. E. Liver fibrosis was assessed by hydroxyproline content, normalized to mass or worm pairs recovered by perfusion of infected mice through the portal vein. Data shown are mean ±SEM and represent two independent experiments (n = 15, ns = not significant).

Intestinal lesions and hepatotoxicity were reported as the pathological features of high dose schistosomiasis in IL-4Rα^flox/Δ^LysM^Cre^ mice [7]. Consequently, we examined the liver and intestine at weeks 9 and 16 post-infection to determine whether similar pathological changes were occurring following infection with 35 cercariae. Strikingly, even after 16 weeks of infection, an experienced pathologist failed to detect any increase in intestinal damage in the IL-4Rα^flox/Δ^LysM^Cre^ group when compared with littermate control mice (**Fig. 2A**), which likely explains the equivalent survival. Liver sections were stained with Giemsa to quantify the granulomatous inflammatory response (**Fig. 2B**) and with picrosirius red to evaluate the accumulation of liver collagen at acute and chronic time points (**Fig. 2C**). Granulomas appeared normally organized in IL-4Rα^flox/Δ^LysM^Cre^ mice with an equivalent proportion of eosinophils, but granuloma size increased significantly compared to littermate controls at both 9 and 16 weeks post-infection (**Fig. 2B and 2D**). Like Herbert *et al*., we found the exacerbated granulomatous inflammation led to only subtle increases in fibrosis, however, and did not lead to statistically significant increases in chronic fibrosis, determined qualitatively by picrosirius red staining (**Fig. 2C**) and quantitatively by hydroxyproline assay (**Fig. 2E**). These observations suggest that while AAMs limit granulomatous inflammation at both acute and chronic time points, additional regulatory mechanisms limit the progression of fibrosis. This interpretation was surprising because uncontrolled granulomatous inflammation in the liver has been hypothesized to contribute to the development of fibrosis in infected mice and humans [12], [13]. These data were also difficult to interpret because Arg1-expressing AAMs are critical to the suppression of fibrosis in infected mice [8], and their numbers should have been greatly diminished in the IL-4Rα^flox/Δ^LysM^Cre^ mice according to Herbert *et al*. and others who have demonstrated Arg1 expression in macrophages is highly dependent on IL-4Rα signaling [7], [14], [15].

### *In vivo* evidence of alternative macrophage activation in IL-4Rα^flox/Δ^LysM^Cre^ mice

**Figure 3:**
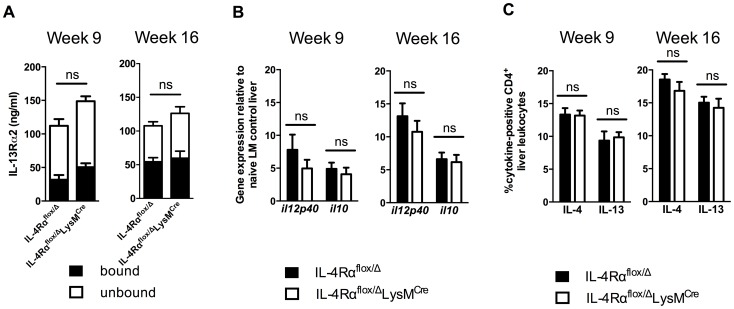
Normal cytokine response in *S. mansoni*-infected IL-4Rα^flox/Δ^LysM^Cre^ mice. IL-4Rα^flox/Δ^LysM^Cre^ mice and IL-4Rα^flox/Δ^ littermate controls were infected percutaneously with 35 *S. mansoni* cercariae. A. Serum IL-13Rα2 levels were measured by ELISA 9 or 16 weeks post-infection. The open and solid portions of each bar correspond to unbound IL-13Rα2 and IL-13Rα2 bound to IL-13, respectively. B. Tissue cytokine levels. Expression of *il12p40* and *il10* was quantified by qPCR from liver tissue snips of IL-4Rα^flox/Δ^LysM^Cre^ mice (open bars) or IL-4Rα^flox/Δ^ littermate controls (solid bars). C. Th2 response. Liver leukocytes were isolated from IL-4Rα^flox/Δ^LysM^Cre^ mice (open bars) or IL-4Rα^flox/Δ^ littermate controls (solid bars), stimulated with phorbol myristate acetate/ionomycin, and analyzed by flow cytometry. The percentage of CD4^+^ leukocytes expressing intracellular IL-4 and IL-13 are shown. (n = 7-15 for each experiment, ns = not significant). Data shown are mean ±SEM and represent two independent experiments.

**Figure 4:**
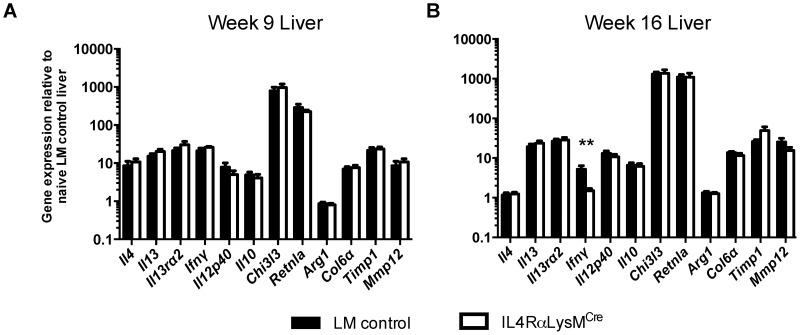
Normal expression of AAM-associated genes in IL-4Rα^flox/Δ^LysM^Cre^ liver. IL-4Rα^flox/Δ^LysM^Cre^ mice (open bars) and IL-4Rα^flox/Δ^ littermate controls (solid bars) were infected percutaneously with 35 cercariae. Expression of selected genes was measured by qPCR in liver tissue 9 weeks (A) and 16 weeks (B) post-infection and normalized to expression in naïve littermate control tissue (n = 7–15; p>0.05 except where noted, **p<0.01). Data shown are mean ±SEM and represent two independent experiments.

Recently, we showed that multiple mechanisms collaborate to slow the progression of fibrosis during chronic schistosome infection [10]. These included IL-13Rα2, a high-affinity decoy receptor for IL-13 [16], [17], IL-12p40, a key driver of Th1 and Th17 responses [18], and IL-10, a potent immunosuppressive cytokine [11], [19]. To determine whether the induction of any of these important immunoregulatory mechanisms was altered in the IL-4Rα^flox/Δ^LysM^Cre^ mice, we analyzed their expression in the granulomatous livers of acutely and chronically infected mice. Levels of IL-13Rα2 in the serum, whether circulating free or bound to IL-13, were indistinguishable between infected IL-4Rα^flox/Δ^ control and IL-4Rα^flox/Δ^LysM^Cre^ mice (**Fig. 3A**). Likewise, IL-12p40 and IL-10 mRNA were expressed at similar levels in the livers of IL-4Rα^flox/Δ^ littermate controls and IL-4Rα^flox/Δ^LysM^Cre^ mice at 9 and 16 weeks post-infection **(** **Fig. 3B** **)**. Expression of both IL-4 and IL-13 by CD4^+^ T cells, the principal stimuli driving fibrosis in this system [20], [21], increased identically at 9 weeks post-infection and remained at equivalent levels through week 16 (**Fig. 3C**). Consistent with the leukocyte responses, we observed no significant increases in IL-4 or IL-13 gene expression in the livers (**Fig. 4**) or intestines (not shown) of IL-4Rα^flox/Δ^LysM^Cre^ mice when compared with expression in IL-4Rα^flox/Δ^ littermate controls 9 and 16 weeks post-infection. These observations suggested a much less critical role for IL-4Rα-expressing AAMs during the chronic response to low dose *S. mansoni* infections.

Surprisingly, however, after further analysis of gene expression in the liver, we found the IL-4Rα^flox/Δ^LysM^Cre^ mice displayed no reduction in the expression of multiple genes that characterize the AAM phenotype [22], including *Chi3l3* (encoding Ym1), *Retnla* (Relm-α), and *Arg1* (**Fig. 4**). Together, the similarities in pathology, survival, and gene expression indicated that in our experiments with IL-4Rα^flox/Δ^LysM^Cre^ mice, AAM development was not substantially impaired or at least not to the degree previously suggested [7], [23].

### A population of IL-4Rα-expressing myeloid cells resisted LysM^Cre^-mediated deletion

**Figure 5:**
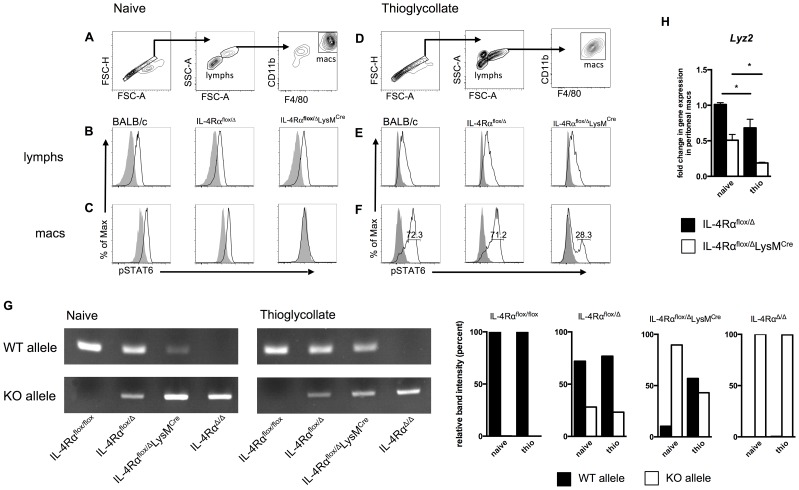
A population of inflammatory IL-4Rα-expressing myeloid cells resists LysM^Cre^-mediated deletion. BALB/c, IL-4Rα^flox/Δ^, and IL-4Rα^flox/Δ^LysM^Cre^ mice were injected i.p. with 2 ml thioglycollate 4 d prior to harvest or were left untreated (naïve). Peritoneal cells were harvested from each group, stimulated for 30 min with 20 ng/ml IL-4 (black outline), and compared to unstimulated cells (solid gray). IL-4Rα function was assessed by IL-4-induced phosphorylation of STAT6 using flow cytometry. A, D. Gating strategy for lymphocytes and F4/80^hi^ CD11b^hi^ macrophages. Detection of pSTAT6 in lymphocytes (B, E) and macrophages (C, F). Each histogram peak represents an individual mouse (n = 2–6 for 2 independent experiments). G. DNA was isolated from F4/80^hi^ CD11b^hi^ macrophages FACS sorted from naïve and thioglycollate-treated IL-4Rα^flox/flox^, IL-4Rα^flox/Δ^, IL-4Rα^flox/Δ^LysM^Cre^ and IL-4Rα^Δ/Δ^ mice. Rearrangement of the *Il4rα* locus was measured using PCR to compare the presence of wild-type (WT) and knockout (KO) *Il4rα* alleles. Quantification of band intensity is shown in the right panels. Aggregate intensity of WT product plus KO product was normalized to 100 percent for each sample. H. In a separate experiment, CD11b^+^ F4/80^+^ macrophages were sorted from the same naïve or thioglycollate-elicited peritoneal cells. *Lyz2* gene expression was found to be lower in thioglycollate-elicited macrophages than naïve macrophages (n = 2–5 for 2 independent experiments). Data shown are mean ±SEM and represent two independent experiments (*p<0.05).

We hypothesized that a subset of myeloid cells in IL-4Rα^flox/Δ^LysM^Cre^ mice resisted LysM^Cre^-mediated gene deletion, remained IL-4Rα-positive, and developed into AAM-like cells with immunoregulatory activity. To test for functional expression of IL-4Rα in different leukocyte populations, we isolated peritoneal cells from naïve controls and IL-4Rα^flox/Δ^LysM^Cre^ mice, stimulated them with IL-4, and measured STAT6 phosphorylation. We used flow cytometry to analyze peritoneal lymphocytes and macrophages separately (**Fig. 5A**). Lymphocytes (**Fig. 5B**) and macrophages (**Fig. 5C**) harvested from wild-type BALB/c and naïve IL-4Rα^flox/Δ^ littermate controls phosphorylated STAT6 to the same degree. Lymphocytes from IL-4Rα^flox/Δ^LysM^Cre^ mice also exhibited normal STAT6 phosphorylation in response to IL-4 (**Fig. 5B**). In contrast, macrophages from naïve LysM^Cre^-expressing mice displayed no STAT6 phosphorylation (**Fig. 5C**), confirming the ablation of IL-4Rα signaling in resident peritoneal macrophages.

Inflammatory immune responses recruit, expand, and replace diverse populations of myeloid cells, and while there is strong evidence that resident tissue macrophages can also expand by proliferating [24], [25], resident cells may become rapidly and greatly outnumbered by monocyte-derived differentiating cells [26]. Therefore, we next examined whether IL-4Rα^flox/Δ^LysM^Cre^ macrophages elicited in response to a sterile inflammatory stimulus are as defective as the resident tissue macrophage population in their response to IL-4. We injected IL-4Rα^flox/Δ^LysM^Cre^ and littermate control mice intraperitoneally (i.p.) with thioglycollate (a stimulus recently shown to elicit only bone-marrow derived inflammatory cells and not to expand tissue resident cells [27]), harvested peritoneal cells 4 days later, and repeated our IL-4-induced phospho-STAT6 assay (**Fig. 5D**). Lymphocytes again displayed similar STAT6 phosphorylation in response to IL-4 (**Fig. 5E**). But critically, over a quarter of the thioglycollate-elicited F4/80^hi^CD11b^hi^ macrophages from IL-4Rα^flox/Δ^LysM^Cre^ mice were still able to respond to IL-4 as shown by phosphorylated STAT6 (**Fig. 5F**). Quantification and statistics are shown in **Fig. S3**. To confirm that this phenomenon is a reflection of genomic rearrangement of the *Il4rα* locus, we isolated DNA from F4/80^hi^CD11b^hi^ macrophages FACS sorted from naïve and thioglycollate-treated IL-4Rα^flox/Δ^LysM^Cre^ mice alongside IL-4Rα^flox/flox^, IL-4Rα^flox/Δ^, and IL-4Rα^Δ/Δ^ controls. As expected, in macrophages sorted from naïve IL-4Rα^flox/Δ^LysM^Cre^ mice, the wild-type (WT) *Il4rα* allele was present minimally compared to the knockout (KO) allele (**Fig. 5G**). In contrast, the WT *Il4rα* allele was markedly more abundant in macrophages taken from thioglycollate-treated IL-4Rα^flox/Δ^LysM^Cre^ mice. Together, these data consistently demonstrate that amongst inflammatory cells in IL-4Rα^flox/Δ^LysM^Cre^ mice, a subset of F4/80^hi^CD11b^hi^ macrophages fails to delete the floxed *Il4rα* gene as expected and therefore remains capable of undergoing IL-4Rα-mediated alternative activation.

We hypothesized that the discrepancy in *Il4rα* excision is explained by differential expression of *Lyz2* (encoding lysozyme M) by the naïve and thioglycollate-elicited peritoneal macrophage populations. We sorted CD11b^hi^F4/80^hi^ macrophages from both peritoneal environments and measured *Lyz2* expression. The magnitude of *Lyz2* expression is lower in IL-4Rα^flox/Δ^LysM^Cre^ mice than corresponding Cre-negative controls because IL-4Rα^flox/Δ^LysM^Cre^ mice transcribe Cre rather than lysozyme M at one locus [28], but in support of the hypothesis, naive macrophages expressed significantly more *Lyz2* than thioglycollate-elicited macrophages in both IL-4Rα^flox/Δ^ and IL-4Rα^flox/Δ^LysM^Cre^ mice (**Fig. 5H**).

### *Lyz2^lo^* macrophages developed features of AAMs in response to *S. mansoni* eggs

**Figure 6:**
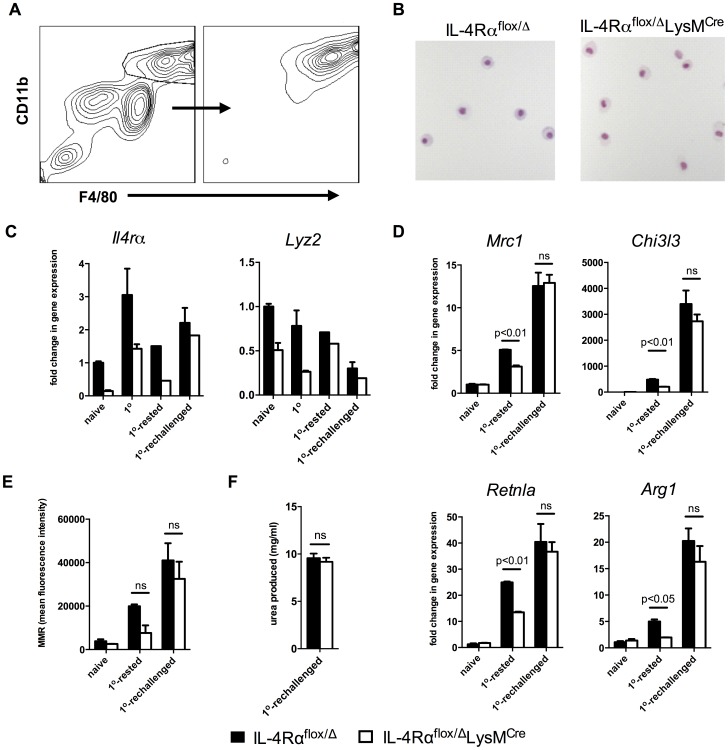
*Lyz2* ^lo^ macrophages develop features of AAMs in response to *S. mansoni* eggs. IL-4Rα^flox/Δ^LysMCre mice (open bars) and littermate controls (solid bars) were left untreated (naïve), challenged with 5000 *S. mansoni* eggs i.p. 4 days before harvest (1^o^), 18 days before harvest (1^o^-rested), or challenged on both 18 days and 4 days before harvest (1^o^-rechallenged). A. Total peritoneal cells were sorted for F4/80^hi^ CD11b^hi^ cells at a purity of >90%. B. Representative 20× images of sorted F4/80^hi^ CD11b^hi^ macrophages after cytospin and hematoylin and eosin staining. C,D. The sorted cells were assayed for *Il4rα* and *Lyz2* gene expression (C), and gene expression of markers of alternative activation (D). Fold change in gene expression is shown relative to the expression levels in sorted F4/80^hi^ CD11b^hi^ cells from naïve littermate controls. E. Surface expression of mannose receptor measured by flow cytometry on unsorted F4/80^hi^ CD11b^hi^ peritoneal cells from the same treatment groups. F. Arginase activity in sorted macrophages. (n = 3–6, ns = not significant) Data shown are mean ±SEM and represent at least two independent experiments.

We next examined whether type-2 response-inducing schistosome eggs also generate a subset of inflammatory macrophages that resists LysM^Cre^-mediated gene deletion. For these studies, we directly compared four distinct populations of peritoneal macrophages: resident macrophages (naïve), *S. mansoni* egg-elicited macrophages 4 days following i.p. egg injection (1^o^), *S. mansoni* egg-elicited macrophages 18 days following i.p. egg injection (1^o^-rested), and macrophages from mice injected i.p. with eggs twice over 14 days and then harvested 4 days after the second challenge (1^o^-rechallenged). F4/80^hi^CD11b^hi^ peritoneal macrophages were sorted from each group (representative flow plots and cytospins in **Fig. 6A–B**; images for each condition are shown in **Fig. S4**), and *Il4rα* and *Lyz2* mRNA expression was quantified by qPCR. Resident peritoneal macrophages isolated from naïve IL-4Rα^flox/Δ^LysM^Cre^ mice had indeed ablated *Il4rα* expression (**Fig. 6C**). In the resident population, IL-4Rα mRNA expression decreased to less than 15% of littermate levels, explaining the absence of IL-4-induced STAT6 phosphorylation, and concurring with the initially reported efficiency of LysM^Cre^-mediated excision [28]. Strikingly, the peritoneal macrophages isolated from IL-4Rα^flox/Δ^LysM^Cre^ mice 4 days after egg challenge (1^o^) expressed *Il4rα* at a level near 50% of littermates. If the macrophages were isolated on day 18 rather than on day 4 after egg challenge (1^o^-rested), more than 50% of the F4/80^hi^CD11b^hi^ macrophages had deleted *Il4rα*, suggesting that maturation in residence or proliferation of resident cells are factors influencing Cre-mediated excision of *Il4rα*. In marked contrast, IL-4Rα^flox/Δ^LysM^Cre^ peritoneal macrophages purified 4 days after the second dose of *S. mansoni* eggs (1^o^-rechallenged) showed no reduction in *Il4rα* expression compared with littermate controls (**Fig. 6C**), suggesting that recently recruited and differentiated F4/80^hi^CD11b^hi^ macrophages had yet to undergo LysM^Cre^-mediated excision. Compared side-by-side, these data suggest that new F4/80^hi^CD11b^hi^ macrophages (through recruitment or proliferation) were most resistant to LysM^Cre^-mediated gene deletion.

As observed with naïve and thioglycollate-elicited macrophages, we hypothesized that differences in expression of *Lyz2* in resident, rested, and recently recruited macrophages explain the pattern of IL-4Rα expression observed in macrophages isolated from the Cre-expressing mice. As expected, resident naive peritoneal macrophages expressed the most *Lyz2* (**Fig. 6C, right panel**). In littermate IL-4Rα^flox/Δ^ mice, *Lyz2* expression by macrophages was between 50–75% as high in the 1^o^ and the 1^o^-rested populations and less than 25% as high in the 1^o^-rechallenged cells (**Fig. 6C,** **right panel**). The greater than 75% reduction in *Lyz2* expression observed in the 1^o^-rechallenged F4/80^hi^CD11b^hi^ macrophages likely explains why the greatest fraction of these cells are resistant to LysM^Cre^-mediated deletion and remain IL-4Rα positive.

Finally, to confirm that inflammatory macrophages in the IL-4Rα^flox/Δ^LysM^Cre^ mice are capable of becoming alternatively activated in response to schistosome eggs *in vivo*, we isolated F4/80^hi^CD11b^hi^ macrophages from the naïve, 1^o^-rested, 1^o^-rechallenged groups and analyzed the gene expression of several well-documented markers of alternative macrophage activation including *Mrc1* (mouse mannose receptor, C type 1), *Chi3l3*, *Retnla*, and *Arg1*. As expected, there was no evidence of alternative activation in the macrophages isolated from naive mice unexposed to schistosome eggs (**Fig. 6D and 6E**). However, consistent with *Il4rα* expression (**Fig. 6C**), F4/80^hi^CD11b^hi^ macrophages isolated from littermate control and IL-4Rα^flox/Δ^LysM^Cre^ 1^o^-rechallenged groups displayed marked and equivalent increases in *Mrc1*, *Chi3l3*, *Retnla*, and *Arg1* mRNA expression (**Fig. 6D**). They also displayed similar cell surface expression of the mannose receptor (**Fig. 6E**) and nearly identical arginase activity (**Fig. 6F**). In contrast, if the egg-elicited macrophages were left two weeks to rest *in vivo*, *Lyz2* mRNA expression was higher (**Fig. 6C**), and the macrophages isolated from the IL-4Rα^flox/Δ^LysM^Cre^ mice expressed lower levels of schistosome egg-induced *Mrc1*, *Chi3l3*, *Retnla*, and *Arg1* mRNA than littermate controls (**Fig. 6D**). Together, these data demonstrate that a substantial population of Arg1-expressing AAMs was preserved in egg-challenged IL-4Rα^flox/Δ^LysM^Cre^ mice, likely explaining why Arg1^flox/flox^Tie2^Cre^ and IL-4Rα^flox/Δ^LysM^Cre^ mice display distinct fibrosis phenotypes following acute and chronic infection with *S. mansoni* [7], [8].

### Myeloid cell populations in livers of *S. mansoni*-infected IL-4Rα^flox/Δ^LysM^Cre^ mice express *Il4rα* and markers of alternative activation

**Figure 7:**
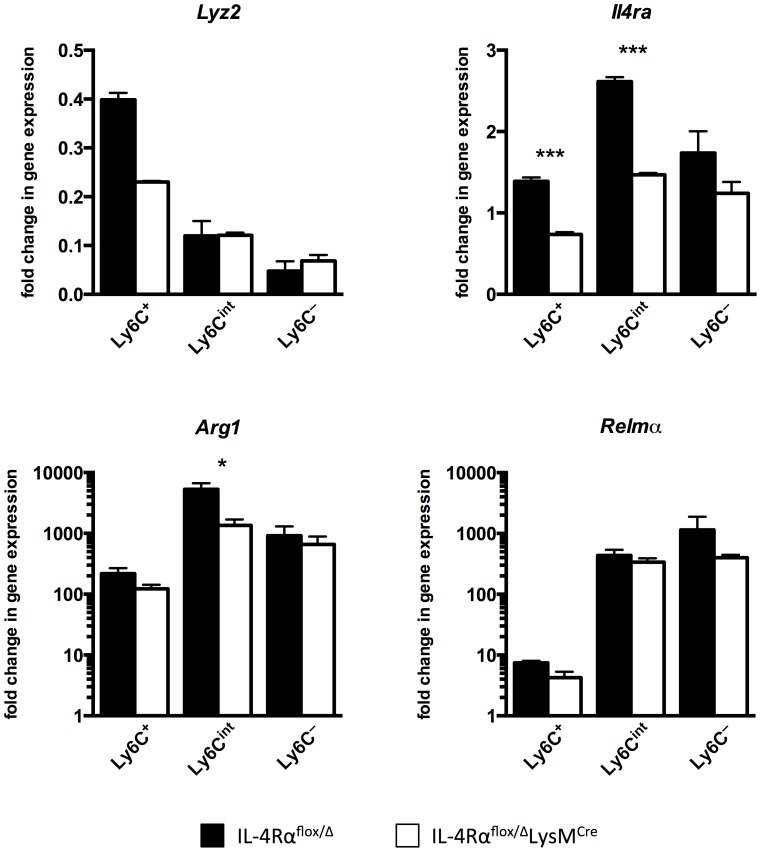
Macrophage populations in livers of *S. mansoni*-infected IL-4Rα^flox/Δ^LysM^Cre^ mice express *Il4r*α and alternative activation markers. IL-4Rα^flox/Δ^LysM^Cre^ mice (open bars) and IL-4Rα^flox/Δ^ littermate controls (solid bars) were infected percutaneously with 35 cercariae. From mice infected for 9 weeks, CD45+ SiglecF- CD11b+ Ly6G- F4/80+ CD64+ liver leukocytes were sorted and separated based on Ly6C expression with a flow cytometer. Gene expression was measured by qPCR (n = 3; *p<0.05, ***p<0.001). Fold change is displayed relative to gene expression from CD45+ SiglecF- CD11b+ Ly6G- F4/80+ CD64+ Ly6C+ cells sorted from naïve IL-4Rα^flox/Δ^ littermate control livers. Data shown are mean ±SEM and represent at least two independent experiments.

Lastly, we sorted myeloid cells from the livers of infected IL-4Rα^flox/Δ^LysM^Cre^ mice and IL-4Rα^flox/Δ^ littermate controls to confirm that there are macrophages resistant to *Il4rα* excision during active infection in the liver and to discern whether *Lyz2* expression was responsible for this. As expected [27], myeloid cells isolated from the infected liver were more heterogeneous than peritoneal macrophages, hence we sorted them as CD45+ SiglecF- CD11b+ Ly6G- F4/80+ CD64+ and then separated them based on Ly6C expression with the aim of gaining insight to their identity as recruited or resident cells (**Fig. S5**). Corroborating observations with peritoneal macrophages exposed to *S. mansoni* eggs, we found that macrophages isolated from infected IL-4Rα^flox/Δ^LysM^Cre^ livers excise *Il4rα* less efficiently than the 83–98% deletion efficiency ascribed to mature macrophages when IL-4Rα^flox/Δ^LysM^Cre^ mice were originally characterized [28] (**Fig. 7**). While *Il4rα* is expressed by all sorted macrophage populations from IL-4Rα^flox/Δ^LysM^Cre^ livers to at least 50% of levels in littermate controls, the Ly6C- cells in particular manifest almost no deficit. Each sorted population also largely maintains alternative activation marker expression. *Arg1* is upregulated several hundred-fold by each sorted population from the infected liver compared to naïve controls although its expression is significantly less in Ly6C^int^ cells from cre-positive mice. Compared to naïve controls, *Relma* expression is greatly upregulated in all sorted populations and at comparable levels in both the groups. Similar results were obtained for *Chi3l3* and *Mrc1* (**Fig. S6**). Furthermore, we observed that the higher the *Lyz2* expression in the sorted macrophage populations, the greater the magnitude of excision of *Il4rα*.

## Discussion

The LysM^Cre^ knock-in mouse has a Cre recombinase gene under control of endogenous lysozyme 2 (*Lyz2*) promoter/enhancer elements and has been used extensively in Cre-lox studies of the myeloid lineage (monocytes, mature macrophages, and granulocytes) for over a decade [28]. In a notable earlier study, conditional IL-4Rα-deficient (IL-4Rα^flox/flox^) mice were crossed to IL-4Rα^Δ/Δ^LysM^Cre^ mice to generate animals with a selective IL-4Rα deletion in macrophages and neutrophils, with the goal of preventing the alternative activation of macrophages [7]. Herbert and colleagues found IL-4Rα^flox/Δ^LysM^Cre^ mice were highly susceptible to acute *S. mansoni* infection (100% mortality by 8 weeks post-infection) because they developed sepsis and severe hepatic and intestinal histopathology. This acute mortality was also associated with increased IFN-γ production and NOS-2 activity, suggesting that AAMs are critically involved in the suppression of highly pathogenic type-1 immune responses during infection with *S. mansoni* [29]. We initiated our studies to directly compare the role of IL-4Rα-deficient and Arg1-deficient macrophages in the pathogenesis of fibrosis [8], but we began to question the merits of this strategy when we discovered IL-4Rα^flox/Δ^LysM^Cre^ mice were not displaying the striking susceptibility to *S. mansoni* infection originally reported by Herbert and colleagues. At a lower dose of 35 cercariae, no difference in mortality occurred between IL-4Rα^flox/Δ^LysM^Cre^ mice and Cre-negative littermates through week 16, and while a larger dose of infectious cercariae accelerated death in both groups, again no difference emerged. It remains difficult to fully explain the difference between the two studies, however the additional controls included in ours, suggest that the global rather than cell-specific deletion of IL-4Rα is the major determinant regulating acute mortality during *S. mansoni* infection. These unexpected results led us to reexamine the role of IL-4Rα-expressing AAMs in the pathogenesis of schistosomiasis.

We first attempted to verify that the mice were indeed deficient in AAMs by isolating peritoneal macrophages from naïve IL-4Rα^flox/Δ^LysM^Cre^ mice and their Cre-negative littermates and stimulating *ex vivo* with IL-4. As expected, STAT6 phosphorylation was entirely defective in IL-4-stimulated macrophages but not in lymphocytes isolated from the IL-4Rα^flox/Δ^LysM^Cre^ mice, confirming myeloid cell-specific deletion of IL-4Rα. However, the livers of infected IL-4Rα^flox/Δ^LysM^Cre^ mice showed little to no reduction in the expression of genes associated with alternative activation [3], suggesting that alternative activation was not significantly impaired *in vivo* during infection.

Most notably, Arg1 mRNA was not reduced, yet Arg1 expression in macrophages is predominantly driven by an IL-4Rα/STAT6-dependent mechanism in schistosomiasis [30]. Arg1 activity was of particular interest because prior studies showed that Arg1-expressing macrophages play a critical host protective role in schistosomiasis by suppressing the pro-inflammatory activity of IL-12/IL-23 during the acute phase and by slowing the progression of IL-13-driven fibrosis in the chronic phase of schistosomiasis [8], [31]. Therefore, we hypothesized that the maintenance of a substantial population of Arg1-expressing AAMs during infection keeps fibrosis and disease progression from being significantly altered in IL-4Rα^flox/Δ^LysM^Cre^ mice, even when chronically infected with *S. mansoni.*

In the original description of *S. mansoni*-infected IL-4Rα^flox/Δ^LysM^Cre^ mice, Herbert and colleagues showed that F4/80^+^ macrophages isolated from the mesenteric lymph nodes of infected mice did not express IL-4Rα, and that peritoneal macrophages from uninfected mice did not respond to IL-4 and IL-13 [7]. Consequently, Arg1 activity was markedly decreased in those macrophages when cultured and stimulated *in vitro*. The behavior of inflammatory macrophages, which dominate most chronic inflammatory diseases remained unknown, however. To begin dissecting the behavior of inflammatory monocytes, we used thioglycollate, a stimulus recently shown to elicit bone marrow-derived inflammatory monocytes but results in nearly undetectable proliferation of tissue resident cells [27]. We compared thioglycollate-elicited peritoneal macrophages with resident peritoneal macrophages. Under this sterile inflammatory condition, over a quarter of the peritoneal macrophage population in IL-4Rα^flox/Δ^LysM^Cre^ mice resisted LysM^Cre^-mediated gene deletion, expressed IL-4Rα, and subsequently developed an Arg1^+^ alternatively activated phenotype when stimulated *ex vivo* with IL-4 or IL-13.

Importantly, when we conducted similar studies eliciting type-2 inflammation by injecting *S. mansoni eggs*, an even greater fraction of the F4/80^hi^CD11b^hi^ macrophage population resisted LysM^Cre^-mediated excision of *Il4rα*. Indeed, if we rechallenged mice with eggs, nearly 100% of the peritoneal macrophages expressed *Il4rα*. As shown recently by Jenkins *et al*., the inflammatory cells could result from proliferation as well as recruitment from monocyte precursors [24], [25]. Mechanistically, we found the peritoneal macrophages in this inflammatory environment also expressed very low levels of *Lyz2*, likely explaining the resistance to LysM^Cre^-mediated excision of *Il4r*α. In addition to driving proliferation of IL-4Rα+ cells, the high levels of IL-4 present following egg exposure can also suppress *Lyz2*, maintaining IL-4Rα expression in LysMCre+ cells [27]. Following rechallenge, the peritoneal macrophages also exhibited an alternatively activated phenotype, with IL-4Rα^flox/Δ^LysM^Cre^ mice and Cre-negative littermates expressing comparably high levels of *Arg1*. However, when the egg-elicited macrophages were given two weeks to mature *in vivo*, a much larger percentage of the isolated macrophages expressed *Lyz2* and deleted *Il4r*α. Nevertheless, even 18 days after *S. mansoni* egg challenge, nearly 40% of the peritoneal macrophages in IL-4Rα^flox/Δ^LysM^Cre^ mice retained IL-4Rαcould respond to IL-4, and exhibited a functional AAM phenotype. Macrophages isolated from the livers of infected IL-4Rα^flox/Δ^LysM^Cre^ mice displayed a similar failure to fully delete the IL-4Rαwhen *Lyz2* expression is lowest, and their AAM phenotype was also preserved. Sorting on Ly6C expression allowed us to distinguish liver macrophage populations with varying levels of *Lyz2* expression, but we were surprised to find the Ly6C- macrophage subset to express the lowest *Lyz2* and be most resistant to *Il4rα* excision. Although Ly6C may be a satisfactory marker for circulating/recently recruited inflammatory monocytes, its regulation during chronic inflammation likely results in more variable expression in the tissue.

Together, our observations of the peritoneum and the infected liver demonstrate that while LysM^Cre^ mice are useful for studying gene function in mature tissue macrophages that have expressed *Lyz2*, they are less effective in chronic disease settings where the resident tissue population is eclipsed by the constant accumulation of immature Lyz2^lo^ macrophages, which in the case of *S. mansoni*-infected IL-4Rα^flox/Δ^LysM^Cre^ mice, remain IL-4Rα^+^ and quickly develop into functional AAMs in response to IL-4 and IL-13 found in the local milieu.

**Figure 8:**
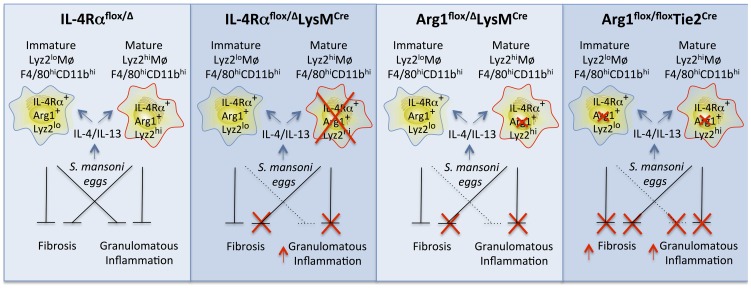
Distinct populations of alternatively activated macrophages control inflammation and fibrosis. Diagram showing the roles of various subsets of alternatively activated macrophages in the pathogenesis of schistosomiasis. In IL-4Rα^flox/Δ^LysM^Cre^ mice, LysM^Cre^ results in the elimination of only mature IL-4Rα^+^Arg1^+^Lyz2^hi^ AAMs. The loss of this subset of AAMs results in the failure to downmodulate granulomatous inflammation in acute and chronic schistosomiasis. However, a substantial population of inflammatory IL-4Rα^+^Lyz2^lo^ AAMs is preserved in these mice that expresses Arg1 in response in response to IL-4 and IL-13 and slows the progression of fibrosis. In Arg1^flox/flox^Tie2^Cre^ mice, both populations of AAMs are defective because of the loss of Arg1 activity, leading to exacerbation of both egg-induced inflammation and fibrosis at both the acute and chronic stage of infection (far right panel). In comparison, Arg1^flox/Δ^LysM^Cre^ primarily deletes Arg1 in the mature Lyz2^hi^ population, leading to a much more modest increase in fibrosis and granulomatous inflammation, which only reaches significance at the chronic stage of infection with *S. mansoni*. Wild-type IL-4Rα^flox/Δ^mice have both mature resident and inflammatory AAMs expressing IL-4Rα and Arg1 so both forms of pathology (inflammation and fibrosis) are substantially controlled in these mice (far left panel).

IL-4Rα^flox/Δ^LysM^Cre^ mice did develop larger granulomas than IL-4Rα^flox/Δ^ littermates at both acute and chronic time points, suggesting the mature Lyz2^hi^ population is the critical subset of AAMs mediating the downmodulation of granulomatous inflammation at the chronic stage of infection. However, these mice did not manifest increased liver fibrosis, portal hypertension, bleeding, or mortality than the littermate controls. Together, these findings were surprising since the progression of fibrosis in schistosomiasis has been linked with the severity of the egg-induced granulomatous response and the ability to downregulate granuloma formation in the chronic phase of the disease [10], [32], [33]. Nevertheless, in studies of *S. mansoni*-infected Arg1^flox/flox^Tie2^Cre^ mice, where Arg1 is deleted from all macrophage populations, fibrosis was substantially increased, suggesting that Arg1 activity in macrophages is critical to the regulation of fibrosis. Thus, we conclude that the preservation of Arg1 activity in more imfmature Lyz2^lo^ F4/80^hi^ CD11b^hi^ macrophages from egg-exposed or infected IL-4Rα^flox/Δ^LysM^Cre^ mice explains why IL-4Rα^flox/Δ^LysM^Cre^ mice, in contrast to Arg1^flox/flox^Tie2^Cre^ mice, did not develop a significantly augmented fibrotic response at any time point [8], [31] (**Summary diagram,** **Fig. 8**). This also likely explains the greater increase in granulomatous inflammation and fibrosis observed in acutely infected Arg1^flox/flox^Tie2^Cre^ versus Arg1^flox/Δ^LysMCre reported previously [8].

Our study demonstrates that a substantial subset of macrophages induced in response to a sterile stimulus or pathogen exposure resists LysM^Cre^-mediated genomic excision. We believe this finding is important because numerous studies have employed the LysM^Cre^ mouse to dissect gene function in macrophages. In some diseases, including gastrointestinal nematode infection and allergic airway disease [7], [34], [35] the reported results could be due to a failure to delete the gene of interest in a sufficient proportion of more immature macrophages arising from proliferation or recruitment from monocyte precursors. We found that while mature “resident” tissue macrophages successfully delete the gene of interest, newly differentiating macrophages in inflammatory environments transcribe insufficient *Lyz2* to efficiently accomplish the Cre-mediated deletion. Our discovery suggests a new experimental approach to distinguish the function of resident tissue and fully mature macrophages from more immature *Lyz2*-negative cells, an emerging topic for research in many infections and inflammatory diseases. Accordingly, our findings complement a recent study showing tissue macrophages and AAMs derived from monocytes are phenotypically distinct [27].

Our findings also demonstrate how quickly this immature *Lyz2* ^lo^ macrophage population can become alternatively activated with high expression of Arg1, which we have shown critically controls the pathogenesis of fibrosis in schistosomiasis [8]. This conclusion is consistent with another recent study that found inflammatory monocytes recruited to the skin quickly adopt a suppressive AAM-like phenotype in response to IL-4 [36]. Collectively, we conclude that it is a Lyz2^lo^ IL-4Rα^+^ Arg1^+^ population of F4/80^hi^CD11b^hi^ macrophages that is critically involved in the suppression of fibrosis in chronic schistosomiasis, while the Lyz2^hi^ IL-4Rα^+^ population of mature resident macrophages controls the magnitude of the egg-induced inflammatory response at both acute and chronic time points post-infection.

## Materials and Methods

### Ethics statement

The National Institute of Allergy and Infectious Diseases Division of Intramural Research Animal Care and Use Program, as part of the National Institutes of Health Intramural Research Program, approved all of the experimental procedures (protocol *LPD 16E*). The Program complies with all applicable provisions of the Animal Welfare Act (http://www.aphis.usda.gov/animal_welfare/downloads/awa/awa.pdf) and other federal statutes and regulations relating to animals.

### Animals

IL-4Rα^flox/Δ^ LysM^WT/Cre^ mice backcrossed on a BALB/c background were kindly provided by Dr. Fred Finkelman (U. Cincinnati, Ohio) and Dr. Frank Brombacher (University of Cape Town; Cape Town, South Africa) [7]. IL-4Rα^flox/flox^ females were crossed with IL-4Rα^Δ/Δ^ LysM^WT/Cre^ males to generate IL-4Rα^flox/Δ^ LysM^WT/Cre^ mice (called IL-4Rα^flox/Δ^LysM^Cre^ in this paper) and Cre-negative IL-4Rα^flox/Δ^ littermates. All cells in both the Cre-positive and Cre-negative mice maintain the *Il4rα* gene on one allele. This breeding scheme prevents embryonic deletion of IL-4Rα by Cre-expressing females. BALB/c and IL-4Rα^Δ/Δ^ mice were obtained from Taconic Farms Inc (Derwood, MD). All animals were housed under specific pathogen-free conditions at the National Institutes of Health in an American Association for the Accreditation of Laboratory Animal Care-approved facility.

### Parasite infection

Mice were infected percutaneously via the tail with 35 or 100 cercariae, as indicated, with a Puerto Rican strain of *Schistosoma mansoni* (NMRI) obtained from infected *Biomphalaria glabrata* snails (Biomedical Research Institute; Rockville, MD). Mice were perfused at the time of euthanasia to determine worm and tissue egg burdens as described previously [37].

### Hematology

Serum was analyzed for liver enzyme quantification at the National Institutes of Health Clinical Center using a Vista Analyzer (Siemens; Deerfield, IL). IL-13Rα2 serum levels were determined by ELISA as previously described [38].

### Histopathology

Liver tissue was fixed in Bouin-Hollande solution, embedded in paraffin for sectioning, and stained (Histopath of America; Clinton, MD) with Wright's Giemsa for analysis of inflammation or picrosirius red for fibrosis analysis. A blinded pathologist measured the size of approximately 30 granulomas in Giemsa-stained sections of each sample. Swiss rolls of small intestine were fixed as above and stained with hematoxylin and eosin for blinded scoring of inflammation.

### Fibrosis assay

Hydroxyproline was measured as a surrogate for collagen content. A known weight of liver tissue was hydrolyzed in 6 N HCl at 110°C for 18 h and then neutralized in 10 N NaOH before colorization. A standard curve comprised of dilutions of 1 mM hydroxyproline (Sigma-Aldrich; St. Louis, MO) was used for quantification [39].

### Hepatic leukocyte isolation for intracellular cytokine staining

About 200 mg of granulomatous liver was ground into a single-cell suspension through a 100-µm nylon mesh. Leukocytes were separated on a 40% Percoll (Sigma-Aldrich) gradient (2000 rpm for 15 min) and treated for 2 min with 1 ml ACK (ammonium chloride–potassium bicarbonate) lysis buffer to lyse erythrocytes. After 3 hours of stimulation with phorbol 12-myristate 13-acetate (PMA 10 ng/ml), ionomycin (1 µg/ml), and Brefeldin A (BFA, 10 µg/ml), leukocytes were fixed and permeabilized for 30 minutes (Cytofix/Cytoperm buffer; BD Biosciences; San Diego, CA) and then stained for 30 minutes with antibodies for CD4 (eBioscience; San Diego, CA), IFN-γ (eBioscience), IL-4 (eBioscience), and IL-13 (eBioscience) diluted in the Permwash buffer (BD Biosciences). Expression of CD4 and the intracellular cytokines was analyzed with a BD FACSCanto II flow cytometer and FlowJo v.7.6 software (Tree Star; Ashland, OR).

### Hepatic leukocyte isolation for sorting of myeloid cells

Whole naïve or granulomatous livers were chopped into fine pieces with a razor blade and digested in 100 units/ml collagenase (Sigma) for 1 hr at 37oC with rocking. The tissue was then ground into a single-cell suspension through a 100-µm nylon mesh. Hepatocytes were pelleted out with a 50 g spin for 5 min for cleaner density separation. Leukocytes were separated on a 40% Percoll (Sigma-Aldrich) gradient (2000 rpm for 15 min) and treated for 2 min with 1 ml ACK (ammonium chloride–potassium bicarbonate) lysis buffer to lyse erythrocytes. Leukocytes were stained for 30 minutes with antibodies for CD16/32 (BDBiosciences), CD45 (Biolegend; San Diego, CA), CD11b (Biolegend), Siglec F (BDBiosciences), Ly6G (BDBiosciences), F4/80 (Biolegend), CD64 (Biolegend), and Ly6C (Biolegend) diluted in FACS buffer. CD45+ SiglecF- CD11b+ Ly6G- F4/80+ CD64+ cells were sorted with at least 90% purity from amongst the stained cells using a FACS Aria (BDBiosciences).

### RNA isolation and quantitative real-time PCR

Liver tissue was homogenized in TRIzol Reagent (Life Technologies; Grand Island, NY) using Precellys 24 (Bertin Technologies; Montigny-le-Bretonneux, France). Total RNA was extracted from the homogenate by addition of chloroform followed by the recommendations of the MagMax-96 Total RNA Isolation Kit (Life Technologies). Total RNA was isolated from peritoneal cells with an RNeasy kit (Qiagen). RNA from all cell types was then reverse transcribed using SuperScript II Reverse Transcriptase (Life Technologies). Real-time RT-PCR was performed on an ABI Prism 7900HT Sequence Detection System (Applied Biosystems). Quantities of mRNA expressed by a particular gene were determined using Power SYBR Green PCR Master Mix (Applied Biosystems), normalized to ribosomal protein, large, P2 (RPLP2) mRNA levels in each sample, and then articulated as a relative increase or decrease compared with mRNA levels expressed by the same gene in uninfected controls. Primers were designed using Primer Express software (version 2.0; Applied Biosystems). Forward and reverse primer sequences are listed in Table S1.

### Peritoneal macrophage isolation

Peritoneal cells were collected by washing the peritoneal cavity with PBS containing 5 mM EDTA. The cells were stained for 30 minutes with anti-mouse antibodies for F4/80 (Biolegend), CD11b (Biolegend), and CD16/32 (BD) diluted in the same buffer. F4/80^hi^CD11b^hi^ cells were sorted with at least 90% purity from amongst the stained cells using a FACS Aria (BDBiosciences).

### *Ex vivo* STAT6 phosphorylation

Mice were intraperitoneally (i.p.) injected with 2 ml 3% thioglycollate (BD; Franklin Lakes, NJ) to elicit peritoneal macrophage recruitment or left untreated, as indicated. 4 days later, peritoneal cells from both groups were harvested as described above, and equal numbers of cells per mouse were resuspended with 20 ng/ml recombinant murine IL-4 (Peprotech) in complete RMPI or complete RMPI alone. The resuspended cells were placed in a 37°C water bath for 30 minutes with periodic agitation. Next, cells were fixed with 1.5% paraformaldehyde, washed, and permeabilized with cold methanol overnight at −20°C. Permeabilized cells were washed twice with PBS containing 0.1% bovine serum albumin and stained with anti-mouse STAT6 (BD), F4/80 (Biolegend), CD11b (eBioscience), Gr1 (BD Pharmingen), and CD16/32 (BD) for 1 hour on a shaker at room temperature. Phosphorylation of STAT6 in F4/80^hi^CD11b^hi^Gr1^–^ macrophages and F4/80^l^°CD11b^lo^Gr1^–^ lymphocytes was measured using a BD FACSCanto II flow cytometer and FlowJo v.7.6 software (Tree Star; Ashland, OR).

### DNA isolation and PCR

To extract DNA, equal numbers of FACS sorted peritoneal cells were resuspended in 25 mM NaOH, incubated at 95°C for 15 minutes, and neutralized with 40 mM Tris-HCl. DNA was amplified with GoTaq DNA Polymerase (Promega) with the following primers: *Il4rα* wild-type: F - 5′-GTACAGCGCACATTGTTTTT-3′, R - 5′-CTCGGCGCACTGACCCATCT-3′; *Il4rα* knockout: F - 5′-GGCTGCCCTGGAATAACC-3′, R - 5′-CCTTTGAGAACTGCGGGCT-3′. Gel was imaged and band intensity quantified with BioSpectrum set up with VisionWorksLS software (UVP; Upland, CA).

### Parasite egg treatment and subsequent macrophage analyses

5000 live *Schistosoma mansoni* eggs (obtained from the same source as the cercariae described above) were injected i.p. to prime some mice on day 0 while others were left untreated. On day 14, some of the primed mice were challenged i.p. with 5000 live eggs, and some of the primed mice were left unchallenged. On day 18, peritoneal cells were harvested from each group of mice (naïve, primed/rested, primed/rechallenged). Equal numbers of unsorted peritoneal cells were stained with anti-mouse antibodies against F4/80 (Biolegend), CD11b (eBioscience), CD16/32 (BD), and CD206 (mouse mannose receptor, C type 1) (Biolegend) or with a rat IgG2a isotype control. CD206 fluorescence on F4/80hiCD11bhi macrophages was measured using a BD FACSCanto II flow cytometer and FlowJo v.7.6 software (Tree Star; Ashland, OR). F4/80hiCD11bhi peritoneal cells were also sorted as described above. Some sorted cells were spun for 5 mins with a Shandon Cytospin 3 centrifuge (Thermo Scientific; Waltham, MA) onto a slide before being fixed with methanol and stained with Diff-Quik (Boehringer). Aliquots of 5×105 sorted cells were resuspended in lysis buffer and arginase activity was measured as previously described [39].

### Statistical analysis

All data were analyzed with Prism (Version 5; GraphPad). Data sets were compared with a two-tailed t-test, and differences were considered significant if *P* values were less than 0.05.

### Accession numbers

*Rplp2*: NM_026020, *Il4*: NM_021283, *Il13*: NM_008355, *Il13rα2*: NM_008356, *Il10*: NM_010548, *Chi3l3*: NM_009892, *Retnla*: NM_020509, *Mrc1*: NM_008625, *Arg1*: NM_007482, *Col6α*: NM_009933, *Timp1*: NM_001044384, *Mmp12*: NM_008605, *Il4rα*: NM_001008700, *Lyz2*: NM_017372, *Ifnγ*: NM_008337, *Il12p40*: NM_008352

## Supporting Information

Figure S1 ***Schistosoma mansoni*** **infection burden is not different between IL-4Rα^flox/Δ^ and IL-4Rα^flox/Δ^LysM^Cre^ mice.** IL-4Rα^flox/Δ^ (solid bars) and IL-4Rα^flox/Δ^LysM^Cre^ mice (open bars) were infected with 35 cercariae and harvested 9 weeks and 16 weeks later. (A) *S. mansoni* worm pairs recovered per mouse from liver perfusion. (B) Number of *S. mansoni* eggs in livers of mice harvested 9 weeks or 16 weeks after infection.
(TIFF)

Figure S2 **IL-4Rα^flox/Δ^LysM^Cre^ mice survive high-dose** ***Schistosoma mansoni*** **infection at the same rate as IL-4Rα^flox/Δ^ littermate controls.** IL-4Rα^flox/Δ^LysM^Cre^ mice (open circles) and IL-4Rα^flox/Δ^ littermate controls (solid circles) were infected percutaneously with 100 *Schistosoma mansoni* cercariae, and survival was monitored for 14 weeks (n = 11–12 per group, ns = not significant).
(TIFF)

Figure S3 **Compilation of pSTAT6 assays of naïve and thioglycollate-elicited peritoneal cells.** As in Figure 5, BALB/c (gray bars), IL-4Rα^flox/Δ^ (solid bars), and IL-4Rα^flox/Δ^LysM^Cre^ (open bars) mice were injected i.p. with 2 ml thioglycollate 4 d prior to harvest or were left untreated (naïve). Peritoneal cells were harvested from each group, stimulated for 30 min with 20 ng/ml IL-4, and compared to unstimulated cells. IL-4Rα function was assessed by measuring IL-4-induced phosphorylation of STAT6 using flow cytometry. The bars represent the percentage of both naïve (A) and thioglycollate-elicited (B) peritoneal lymphocytes and F4/80^hi^ CD11b^hi^ macrophages phosphorylating STAT6 following IL-4 stimulation. Data shown are mean ± SEM and represent at least two independent experiments (n = 2–6).
(TIFF)

Figure S4 **Flow sorting strategy for** ***S. mansoni*** **egg-induced peritoneal macrophages.** IL-4Rα^flox/Δ^LysMCre mice and littermate controls were left untreated (naïve), challenged with 5000 *S. mansoni* eggs i.p. 4 days before harvest (1^o^), 18 days before harvest (1^o^-rested), or challenged on both 18 days and 4 days before harvest (1^o^-rechallenged). Total peritoneal cells were collected from mice in each treatment group. The cells were sorted for F4/80^hi^ CD11b^hi^ cells at a purity of >90% (left panels). Representative 20× images of sorted F4/80^hi^ CD11b^hi^ macrophages after cytospin and hematoylin and eosin staining are shown in panels on the right.
(TIFF)

Figure S5 **Flow sorting strategy for isolation of macrophage populations from** ***S. mansoni*** **-infected livers.** IL-4Rα^flox/Δ^LysM^Cre^ mice and IL-4Rα^flox/Δ^ littermate controls were infected percutaneously with 35 cercariae. From mice infected for 9 weeks, liver leukocytes were isolated as described in Experimental Procedures. Using flow cytometry, cells were selected for sorting by first gating cells that were live, followed by singlets, CD45+ CD11b+, SiglecF-, and Ly6G-. Ly6G- cells were gated by Ly6C expression, and finally, F4/80+ CD64+ Ly6C-, F4/80+ CD64+ Ly6Cint, and F4/80+ CD64+ Ly6C+ cells were collected at >90% purity for qPCR analysis. Cytospins resulted in thinly dispersed cells so 100× images of individual macrophages are shown below that are representative of the collected populations.
(TIFF)

Figure S6 **Myeloid cell populations in livers of** ***S. mansoni*** **-infected IL-4Rα^flox/Δ^LysM^Cre^ mice express** ***Chi3l3*** **and** ***Mrc1*** **.** IL-4Rα^flox/Δ^LysM^Cre^ mice (open bars) and IL-4Rα^flox/Δ^ littermate controls (solid bars) were infected percutaneously with 35 cercariae. 9 weeks post-infection, CD45+ SiglecF- CD11b+ Ly6G- F4/80+ CD64+ liver leukocytes were sorted and separated based on Ly6C expression with a flow cytometer. Gene expression was measured by qPCR (n = 3; **p<0.01). Fold change is displayed relative to gene expression from CD45+ SiglecF- CD11b+ Ly6G- F4/80+ CD64+ Ly6C^int^ cells sorted from infected IL-4Rα^flox/Δ^ littermate control livers. Data shown are mean ±SEM and represent at least two independent experiments.
(TIFF)

Table S1 **qPCR primer sequences.**
(DOCX)
