# Supplementary figures and images for: Incomplete Deletion of IL-4Rα by LysMCre Reveals Distinct Subsets of M2 Macrophages Controlling Inflammation and Fibrosis in Chronic Schistosomiasis
Source: PLoS Pathog. 2014 Sep 11;10(9):e1004372. doi: 10.1371/journal.ppat.1004372 (PMC4161449; doi:10.1371/journal.ppat.1004372)

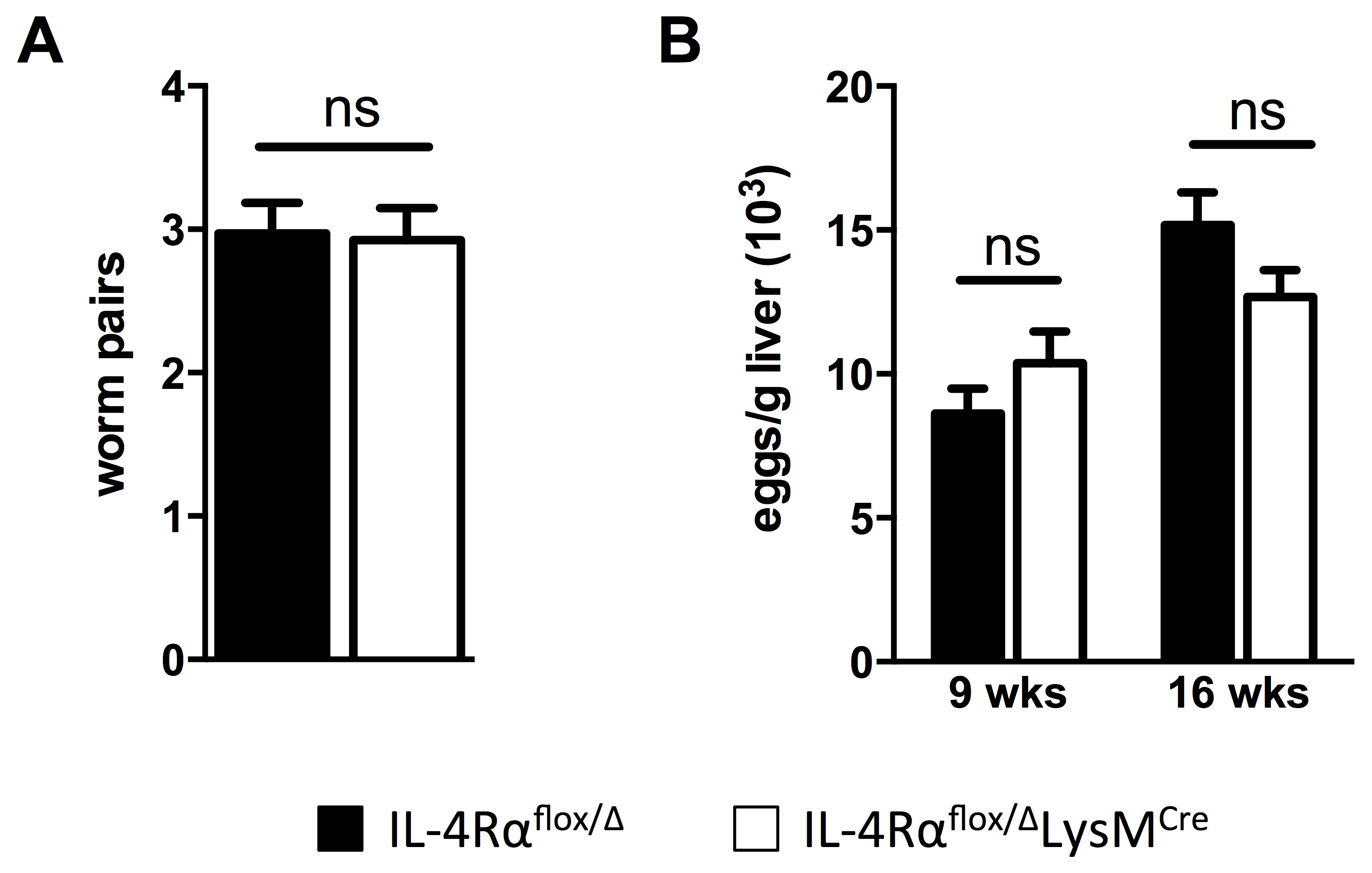

Supplement: Figure S1 — Schistosoma mansoni infection burden is not different between IL-4Rαflox/Δ and IL-4Rαflox/ΔLysMCre mice. IL-4Rαflox/Δ (solid bars) and IL-4Rαflox/ΔLysMCre mice (open bars) were infected with 35 cercariae and harvested 9 weeks and 16 weeks later. (A) S. mansoni worm pairs recovered per mouse from liver perfusion. (B) Number of S. mansoni eggs in livers of mice harvested 9 weeks or 16 weeks after infection. (TIFF) [file ppat.1004372.s001.tiff]

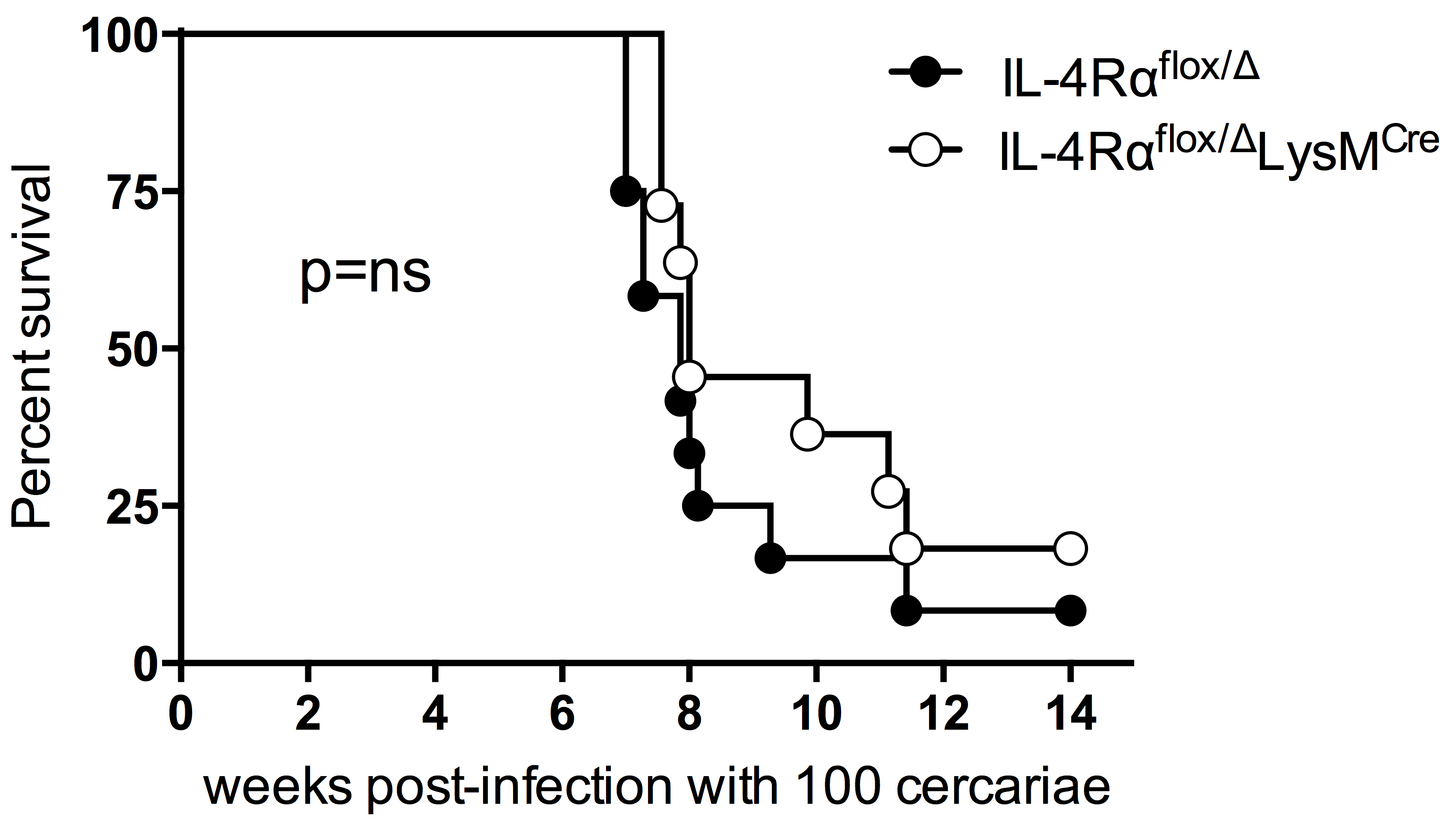

Supplement: Figure S2 — IL-4Rαflox/ΔLysMCre mice survive high-dose Schistosoma mansoni infection at the same rate as IL-4Rαflox/Δ littermate controls. IL-4Rαflox/ΔLysMCre mice (open circles) and IL-4Rαflox/Δ littermate controls (solid circles) were infected percutaneously with 100 Schistosoma mansoni cercariae, and survival was monitored for 14 weeks (n = 11–12 per group, ns = not significant). (TIFF) [file ppat.1004372.s002.tiff]

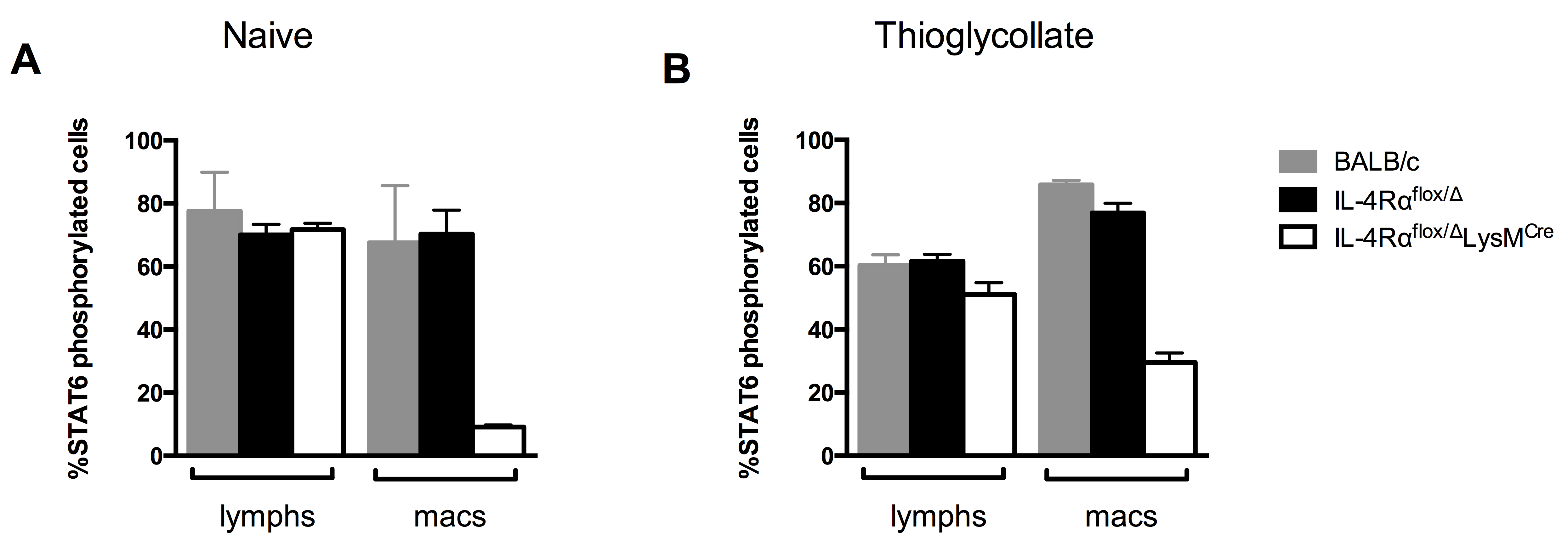

Supplement: Figure S3 — Compilation of pSTAT6 assays of naïve and thioglycollate-elicited peritoneal cells. As in Figure 5, BALB/c (gray bars), IL-4Rαflox/Δ (solid bars), and IL-4Rαflox/ΔLysMCre (open bars) mice were injected i.p. with 2 ml thioglycollate 4 d prior to harvest or were left untreated (naïve). Peritoneal cells were harvested from each group, stimulated for 30 min with 20 ng/ml IL-4, and compared to unstimulated cells. IL-4Rα function was assessed by measuring IL-4-induced phosphorylation of STAT6 using flow cytometry. The bars represent the percentage of both naïve (A) and thioglycollate-elicited (B) peritoneal lymphocytes and F4/80hi CD11bhi macrophages phosphorylating STAT6 following IL-4 stimulation. Data shown are mean ± SEM and represent at least two independent experiments (n = 2–6). (TIFF) [file ppat.1004372.s003.tiff]

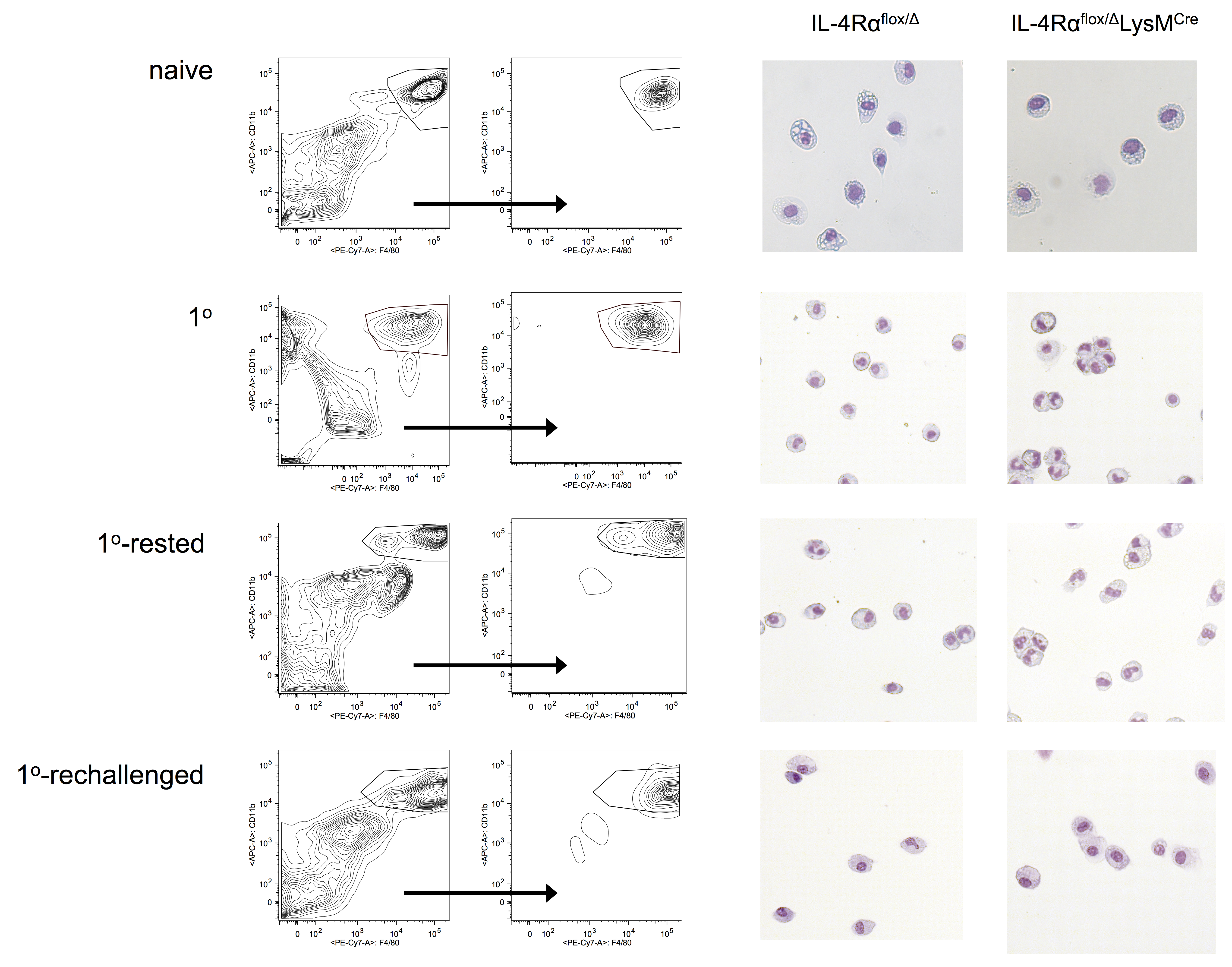

Supplement: Figure S4 — Flow sorting strategy for S. mansoni egg-induced peritoneal macrophages. IL-4Rαflox/ΔLysMCre mice and littermate controls were left untreated (naïve), challenged with 5000 S. mansoni eggs i.p. 4 days before harvest (1o), 18 days before harvest (1o-rested), or challenged on both 18 days and 4 days before harvest (1o-rechallenged). Total peritoneal cells were collected from mice in each treatment group. The cells were sorted for F4/80hi CD11bhi cells at a purity of >90% (left panels). Representative 20× images of sorted F4/80hi CD11bhi macrophages after cytospin and hematoylin and eosin staining are shown in panels on the right. (TIFF) [file ppat.1004372.s004.tiff]

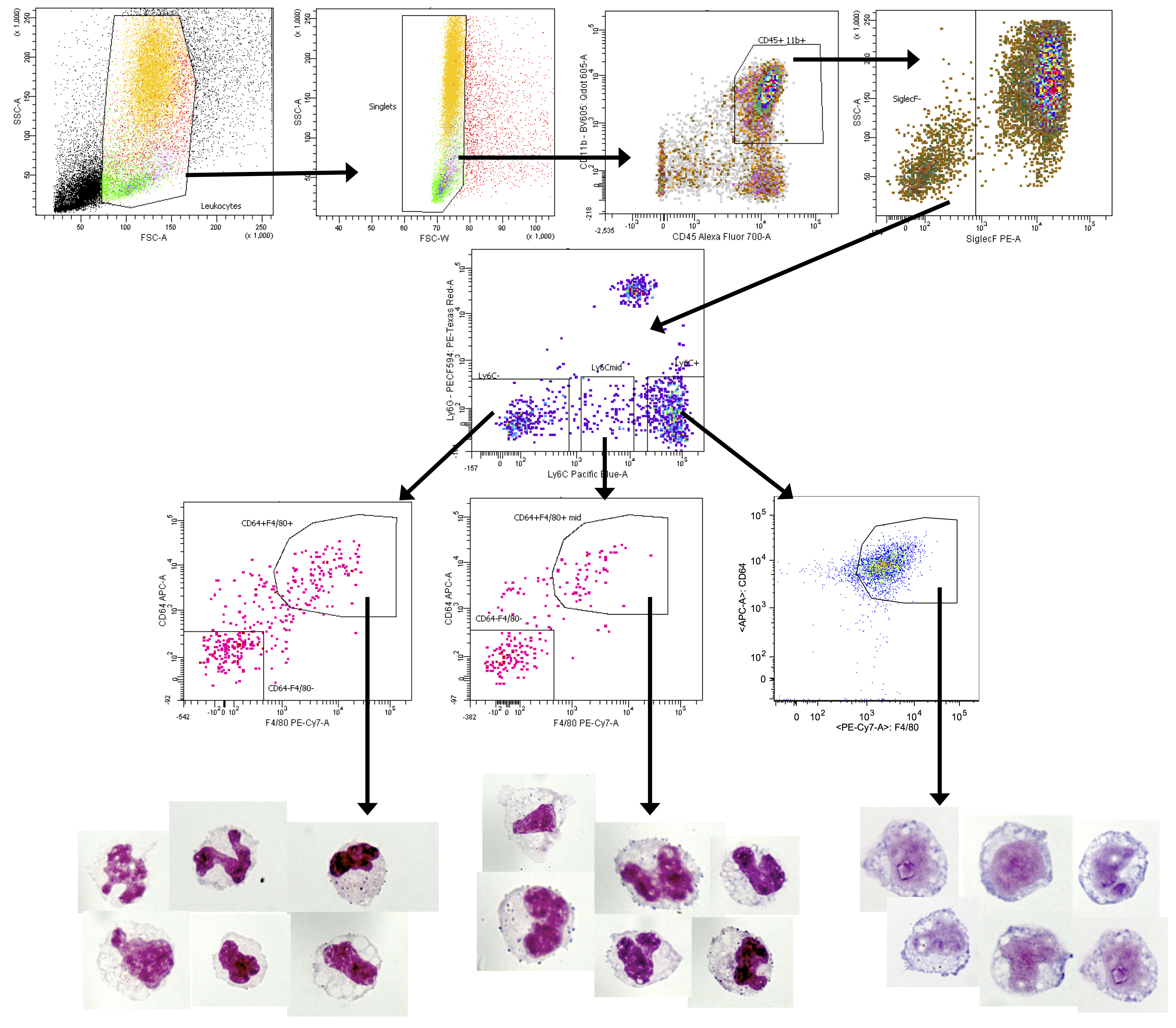

Supplement: Figure S5 — Flow sorting strategy for isolation of macrophage populations from S. mansoni -infected livers. IL-4Rαflox/ΔLysMCre mice and IL-4Rαflox/Δ littermate controls were infected percutaneously with 35 cercariae. From mice infected for 9 weeks, liver leukocytes were isolated as described in Experimental Procedures. Using flow cytometry, cells were selected for sorting by first gating cells that were live, followed by singlets, CD45+ CD11b+, SiglecF-, and Ly6G-. Ly6G- cells were gated by Ly6C expression, and finally, F4/80+ CD64+ Ly6C-, F4/80+ CD64+ Ly6Cint, and F4/80+ CD64+ Ly6C+ cells were collected at >90% purity for qPCR analysis. Cytospins resulted in thinly dispersed cells so 100× images of individual macrophages are shown below that are representative of the collected populations. (TIFF) [file ppat.1004372.s005.tiff]

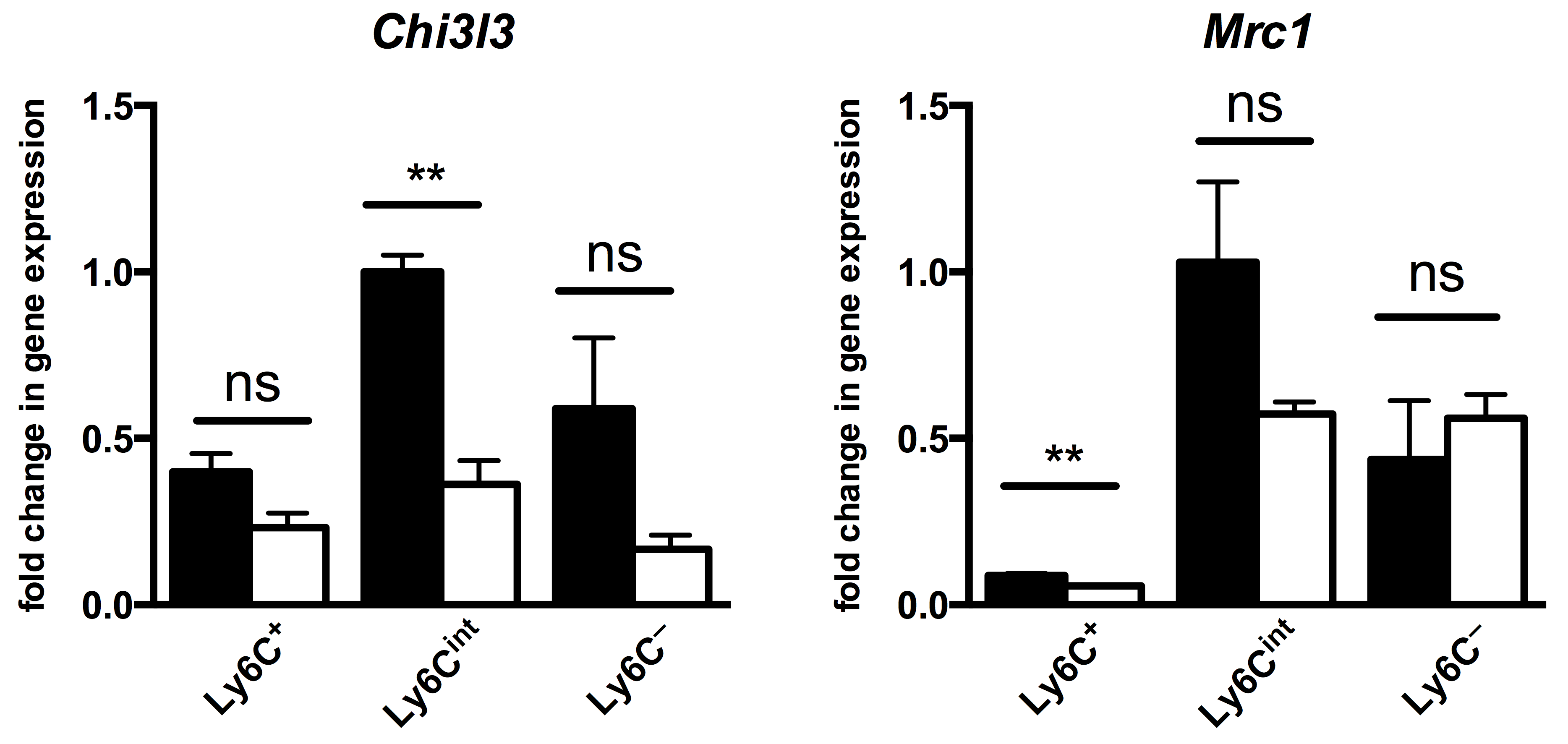

Supplement: Figure S6 — Myeloid cell populations in livers of S. mansoni -infected IL-4Rαflox/ΔLysMCre mice express Chi3l3 and Mrc1 . IL-4Rαflox/ΔLysMCre mice (open bars) and IL-4Rαflox/Δ littermate controls (solid bars) were infected percutaneously with 35 cercariae. 9 weeks post-infection, CD45+ SiglecF- CD11b+ Ly6G- F4/80+ CD64+ liver leukocytes were sorted and separated based on Ly6C expression with a flow cytometer. Gene expression was measured by qPCR (n = 3; **p<0.01). Fold change is displayed relative to gene expression from CD45+ SiglecF- CD11b+ Ly6G- F4/80+ CD64+ Ly6Cint cells sorted from infected IL-4Rαflox/Δ littermate control livers. Data shown are mean ±SEM and represent at least two independent experiments. (TIFF) [file ppat.1004372.s006.tiff]
