# Supplementary material for: Incomplete Deletion of IL-4Rα by LysMCre Reveals Distinct Subsets of M2 Macrophages Controlling Inflammation and Fibrosis in Chronic Schistosomiasis
Source: PLoS Pathog. 2014 Sep 11;10(9):e1004372. doi: 10.1371/journal.ppat.1004372 (PMC4161449; doi:10.1371/journal.ppat.1004372)
Supplement: Table S1 — qPCR primer sequences. (DOCX) [file ppat.1004372.s007.docx]

**Table S1. qPCR Primer Sequences.**

| Gene | Forward qPCR primer sequence | Reverse qPCR primer sequence |
| --- | --- | --- |
| *Rplp2* | TACGTCGCCTCTTACCTGCT | GACCTTGTTGAGCCGATCAT |
| *Il4* | ACGAGGTCACAGGAGAAGGGA | AGCCCTACAGACGAGCTCACTC |
| *Il13* | CCTCTGACCCTTAAGGAGCTTAT | CGTTGCACAGGGGAGTCT |
| *Il13rα2* | GGAAAGGAGGACAAAGAGGTC | GATTTAGTGTGCTGAAAGCTCTACTC |
| *Il10* | ATGCTGCCTGCTCTTACTGACTG | CCCAAGTAACCCTTAAAGTCCTGC |
| *Chi3l3* | CATGAGCAAGACTTGCGTGAC | GGTCCAAACTTCCATCCTCCA |
| *Retnla* | CCCTCCACTGTAACGAAGACTC | CACACCCAGTAGCAGTCATCC |
| *Mrc1* | CCCAAGGGCTCTTCTAAAGCA | CGCCGGCACCTATCACA |
| *Arg1* | GGAAAGCCAATGAAGAGCTG | GCTTCCAACTGCCAGACTGT |
| *Col6α* | CGCCCTTCCCACTGACAA | GCGTTCCCTTTAAGACAGTTGAG |
| *Timp1* | GCAACTCGGACCTGGTCATAA | CGGCCCGTGATGAGAAACT |
| *Mmp12* | AATGCTGCAGCCCCAAGGAAT | CTGGGCAACTGGACAACTCAACTC |
| *Il4rα*^a^ | GAGTGGAGTCCTAGCATCACG | CAGTGGAAGGCGCTGTATC |
| *Lyz2* | TGGGATCAATTGCAGTGCT | CACCACCCTCTTTGCACATT |
| *Ifnγ* | AGAGCCAGATTATCTCTTTCTACCTCAG | CCTTTTTCGCCTTGCTGTTG |
| *Il12p40* | TGGTTTGCCATCGTTTTGCTG | ACAGGTGAGGTTCACTGTTTCT |

^a^ floxed region in exons 7 and 8
